# CO_2_ Laser Surgery and Prosthetic Management for the Treatment of Epulis Fissuratum

**DOI:** 10.5402/2011/282361

**Published:** 2010-11-28

**Authors:** Tarcisio José de Arruda Paes-Junior, Sâmia Carolina Mota Cavalcanti, Daniela Fernandes Figueira Nascimento, Guilherme de Siqueira Ferreira Anzaloni Saavedra, Estevão Tomomitsu Kimpara, Alexandre Luiz Souto Borges, Walter Niccoli-Filho, Paula Carolina de Paiva Komori

**Affiliations:** ^1^Department of Dental Materials and Prosthodontics, São Jose dos Campos Dental School, São Paulo State University, São José dos Campos, Avenida Francisco José Longo, 777 Vila Adyana, 12245-000 São Jose dos Campos, Brazil; ^2^Department of Biopathology and Diagnosis and Academic Group of Studies and Research with Lasers in Dentistry, São Jose dos Campos Dental School, São Paulo State University, 12245-000 São José dos Campos, Brazil

## Abstract

The aim of this study was to present a case report of the surgical removal of hyperplasia in the oral cavity, using carbon dioxide (CO_2_) laser radiation and rehabilitation with a complete denture. *Epulis fissuratum* occurs in complete denture patients, because a constant irritative action induces the mucosa to grow under poorly fitting dentures. These lesions must be removed, and to avoid a relapse, new complete dentures should be made to maintain healthy surgical tissues. The clinical sequence presented in this case shows a completely edentulous patient with *epulis fissuratum* on the lower alveolar ridge extending to the vestibular sulcus of the anterior region of mandible. Immediate complete dentures were made prior to the lesion removal with CO_2_ laser radiation, providing satisfactory results in oral function and tissue health.

## 1. Introduction

Hyperplasia refers to tissue growth into the oral cavity, located over the alveolar ridges or the soft tissues of the vestibular sulcus. Its etiology is multifactorial, but some irritative factors are more commonly associated, such as periodontal disease, poor oral hygiene, smoking, and poor denture fitting [1].

The treatment of this kind of lesion includes elimination of the causing factors and surgical removal of the lesion. If the causal factor persists, the tissue becomes more fibrous over time [2, 3]. The most common techniques used for removing the hyperplastic lesion are surgical scalpel, electrical scalpel, carbon dioxide laser, Erbium: YAG laser, Neodymium: YAG laser, and diode laser [1].

The CO_2_ laser is an appropriate option for surgical procedures in soft tissues since it operates at a wavelength of 10.6 n*μ*, which is within the medium range of the electromagnetic infrared spectrum. This wavelength is absorbed by tissues with high water content [4, 5]. This energy is transformed into heat, causing cell rupture from water boiling [5]; therefore, tissues with high water content suffer less damage.

Many advantages of the CO_2_ laser include the possibility of minimal bleeding, decreasing edemas, flexibility of the wound healing tissue, reduced postoperative pain, and no need of a conventional suture [6–10]. These positive aspects of the use of a CO_2_ laser has allowed an improvement in maxillofacial surgeries.

Despite some disadvantages of CO_2_ laser, such as the delay in the initial repair chronology due to heat necrosis [11, 12], this technique provides suitable repair without scar formation and constitutes an alternative to the conventional incision and suture method.

## 2. Case Report

**Figure 1:**
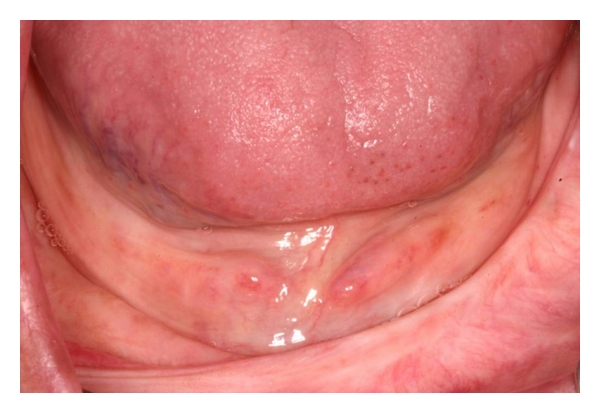
*Epulis fissuratum *over the alveolar ridge extending to the vestibular sulcus in the lower anterior region.

**Figure 2:**
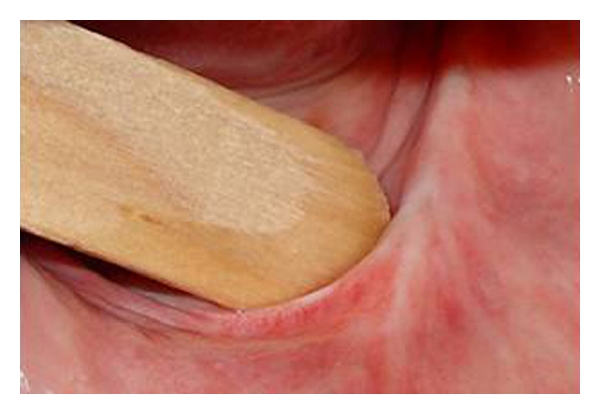
Closer view of *Epulis fissuratum. *

A sixty-three-year Caucasian old female presented at the Dental Clinic of the University (São Jose dos Campos Dental School—UNESP) looking for dental treatment. The clinical exam showed completely edentulous and presented a hyperplastic lesion over the alveolar ridge extending to the vestibular sulcus in the lower anterior region (Figures 1 and 2). According to the patient, two previous surgeries were performed to remove the excess of mucosa, but it relapsed twice. This lesion could impair the retention and stability of a future prosthesis. Considering this clinical situation and the relapse history, the immediate confection of dentures and surgical removal with CO_2_ laser radiation was proposed.

**Figure 3:**
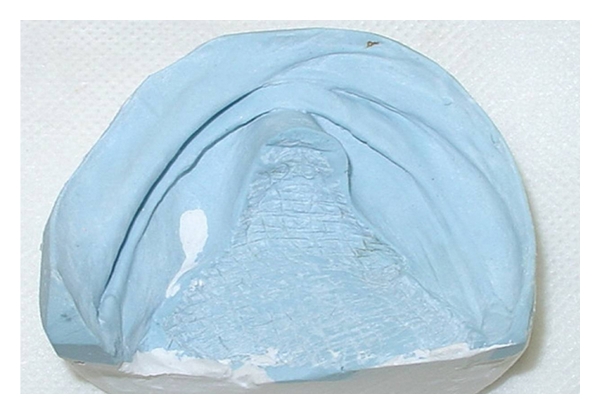
Cast of the lower arch obtained from irreversible hydrocolloid impression.

**Figure 4:**
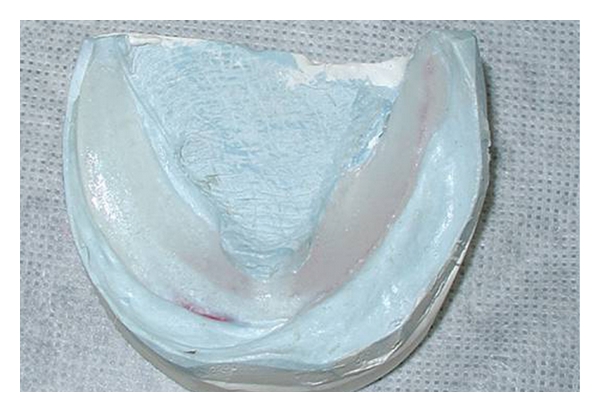
Experimental bases were made outlining the lesion area of the cast.

**Figure 5:**
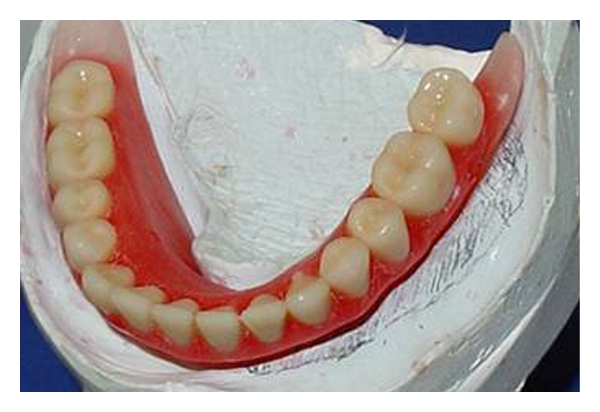
Wax ridges were made over the experimental bases and teeth were mounted following the wax ridges patterns.

The procedures for the confection of a complete denture consisted of a single impression of the upper and lower arch with an irreversible hydrocolloid to record the ridge and lesion area. Casts were obtained (Figure 3), and experimental bases (Figure 4) with wax ridges were made, outlining the lesion area. Compensating curves were registered, and the wax ridges were fixed in centric relation. These casts were then mounted in an arcon semiadjustable articulator. The teeth were mounted following the wax ridges (Figure 5) and aesthetic approval was received from the patient.

**Figure 6:**
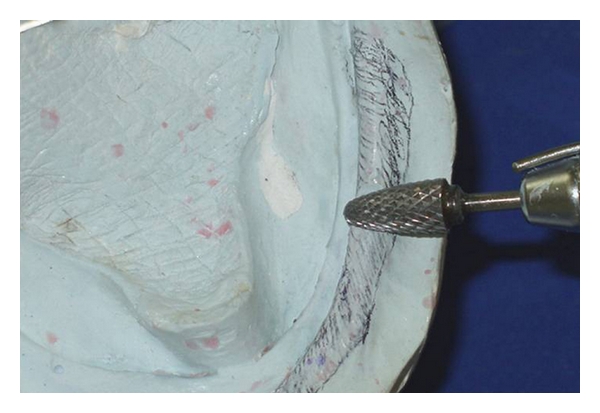
The lesion area was outlined at the lower cast and removed with a burr to the extension of the denture base.

**Figure 7:**
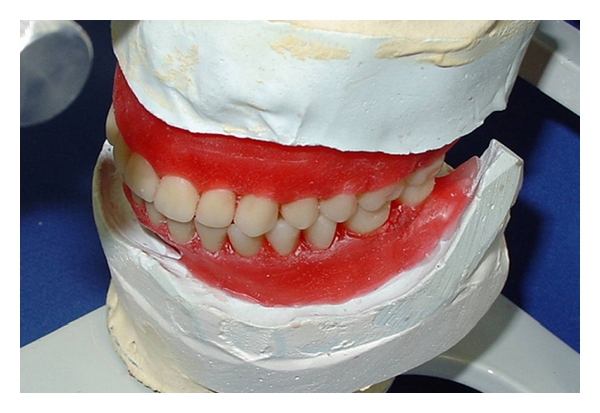
The removed area of the cast correspondent to the lesion was covered with wax.

**Figure 8:**
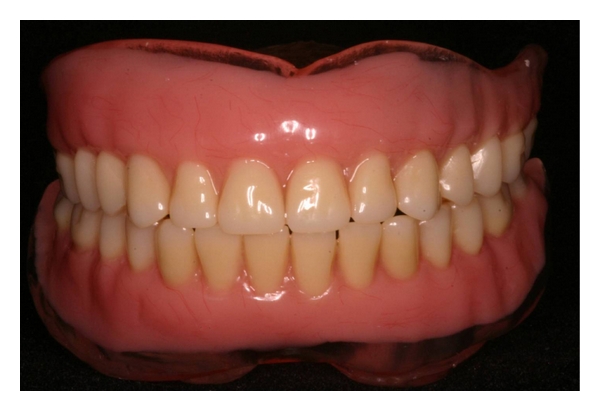
Both the upper and lower dentures were polymerized.

**Figure 9:**
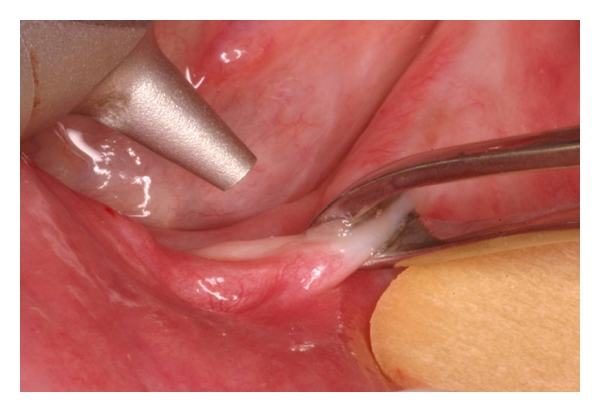
Application of the CO_2_ laser at the lesion.

**Figure 10:**
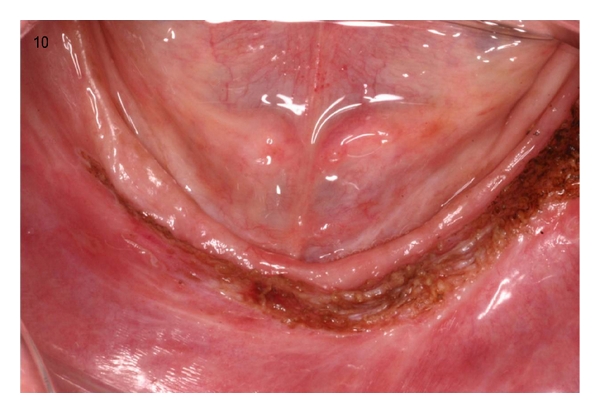
The aspect of the surgical area after the use of CO_2_ laser.

After an adequate aesthetic result was obtained, the lesion area outlined at the lower cast was removed with a burr (Figure 6) to the extension of the denture base. This area of the cast was covered with wax (Figure 7), and both the upper and lower dentures were cured (Figure 8). Denture manufacture was completed and the surgery proceeded. The lesion removal was made using a carbon dioxide laser (Sharplan 15 F, Israel-FAPESP 97/07645-2), and to carry out the vaporization the CO_2_ laser was used in continuous mode with a focused beam with diameter of 4 mm and 8 watt (Figure 9) the vaporized area was clinically evaluated to verify the absence of bleeding (Figure 10). Safety glasses, masks, and procedure gloves protected the professionals who participated in the surgery, and vaporization was done under constant aspiration of the plume. 

**Figure 11:**
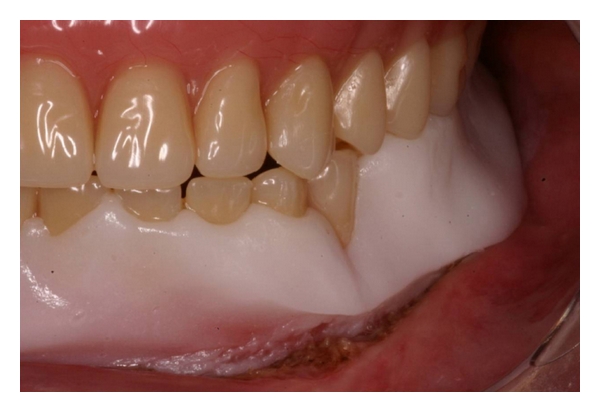
The lower denture was rebased with a soft tissue conditioner to allow adequate tissue repair.

Dentures were positioned, and the lower denture was immediately rebased with a soft tissue conditioner to stabilize the denture and facilitate wound healing (Figure 11).

**Figure 12:**
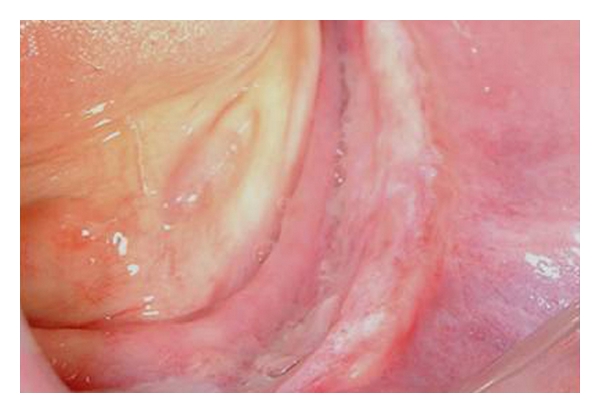
The aspect of the postsurgical area after 7 days.

**Figure 13:**
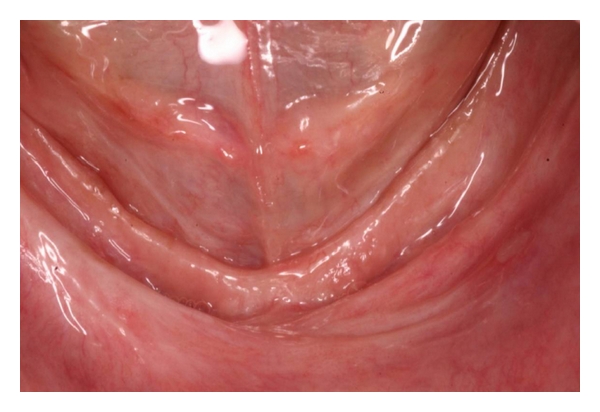
The aspect of the post surgical area after 30 days.

Postoperative examination was done after seven days and showed satisfactory tissue repair at the surgical area (Figure 12). The postsurgical exams were done after 7, 14, 21, and 30 days (Figure 13) of continual wearing of the prosthesis.

## 3. Discussion

The clinical results of the case report presented are in agreement with other studies utilizing CO_2_ laser for soft tissues.

In the current study, the CO_2_ laser was used in continuous mode to control the extension of thermal damage using a focused beam to reduce its intensity. Kauvar et al. [13] histologically demonstrated that the CO_2_ laser, in both the pulse and continuous modes, allowed superficial ablation with minimal thermal damage. Some authors, like Dobry et al. [14], also affirmed that the pulse mode caused less thermal damage, but that a longer actuation time was needed with the tissue.

The advantages of using a CO_2_ laser has been clinically demonstrated in the present study, presenting minimal bleeding during the surgery with no need of sutures while also presenting a good healing response, with minimal wound contraction, less inflammatory reaction, and good re-epithelialization with no scar formation. These conditions were also found in the studies of de Arruda Paes and Niccoli-Filho [12] and Keng and Loh [15] who clinically demonstrated that CO_2_ laser is ideal for use in these kind of surgeries, bringing comfort and aesthetics to the patient. Luomanen et al. [16] explained that less wound contraction occurs because the CO_2_ laser does not remove the tissue collagen. Kardos et al. [17] showed that CO_2_ laser is effective in vaporizing oral mucosa and achieving haemostasis by vessel coagulation.

No hemorrhaging episodes or infections occurred during the postoperative exams. These findings are in agreement with Niccoli-Filho et al. [9] who also demonstrated that good aesthetic and functional characteristics were quickly achieved in an oral surgery using CO_2_ laser, enabling an earlier prosthetic rehabilitation. Thus, the CO_2_ laser is a useful instrument, which provides control of the surgical field and esthetic and functional results [18]. The technique presented in this paper was easily executable and allowed a better prediction of the surgery results.

## 4. Conclusion

Based on the results of the present case report we can conclude:

1. the use of a CO_2_ laser radiation allowed good haemostasis at the surgical area, absence of infections and post operative comfort;
2. the use of CO_2_ laser radiation to remove hyperplastic tissue make the use of prosthesis feasible and promoted an immediately condition to reestablish the aesthetic and functional aspects.
